# B-mode sonographic evaluation of optic nerve sheath diameter and lens thickness in Nigerian adults with glaucoma

**DOI:** 10.4314/ahs.v18i2.19

**Published:** 2018-06

**Authors:** Achimugu G Omatiga, Oluwatoyin H Onakpoya, Bukunmi M Idowu, Christianah M Asaleye, Bernice O Adegbehingbe, Adeniyi S Aderibigbe

**Affiliations:** 1 Department of Radiology, Obafemi Awolowo University Teaching Hospitals Complex, Ile-Ife, Osun State, Nigeria; 2 Department of Ophthalmology, Obafemi Awolowo University Teaching Hospitals Complex, Ile-Ife, Osun State, Nigeria

**Keywords:** Glaucoma, ultrasonography, optic nerve sheath diameter, lens thickness

## Abstract

**Objective:**

This study was done to investigate the effect(s) of glaucoma on the ocular optic nerve sheath diameter and lens thickness using B-mode ultrasonography.

**Materials and methods:**

One hundred and twenty study participants were recruited; 60 subjects with glaucoma and 60 age- and sex-matched controls without glaucoma. The optic nerve sheath diameter and lens thickness of both eyes were measured using a linear high frequency transducer with frequency of 6.5–12MHz.

**Results:**

The mean optic nerve sheath diameter of the glaucomatous eyes (3.57 ± 0.19mm and 3.59 ± 0.33mm on the right and left, respectively) were significantly thinner than that of controls (4.23 ± 0.34 mm and 4.26 ± 0.30 mm on the right and left, respectively; p < 0.001). There is increased mean lens thickness in the glaucomatous eyes (4.15 ± 0.43mm and 4.18 ± 0.46mm on the right and left, respectively) than in the controls (4.01 ± 0.56mm and 3.99 ± 0.45mm on the right and left, respectively) with a statistically significant difference seen in the left eye (p = 0.024).

**Conclusion:**

B-mode ultrasound is a reliable tool of assessing the nerve sheath diameter and lens thickness in glaucoma. Optic nerve sheath diameter is reduced in glaucoma

## Introduction

Glaucoma is a major disease of public health importance being one of the leading causes of blindness worldwide. The estimated prevalence of glaucoma in our sub-region is about 4% in people aged 40 years and above.^1^

Glaucoma is classified into primary, secondary and developmental types. In primary glaucoma, no underlying cause is found for the elevated intraocular pressure (IOP); in secondary glaucoma, the elevated IOP results from other ocular diseases, systemic diseases, or drug use.^2^ Elevated IOP in developmental glaucoma results from embryonic developmental anomalies in the anterior chamber angle. Primary glaucoma is further sub-divided into primary open-angle glaucoma (POAG), which encompasses both conventional POAG and normal-tension glaucoma, and primary closed-angle glaucoma.^2^ POAG is the predominant type in our environment.^3^

In POAG, flow of aqueous humour through the trabecular meshwork is diminished due to the degeneration and obstruction of the trabecular meshwork, whose original function is to absorb the aqueous humour. The reduced aqueous humour outflow leads to increased resistance and thus a chronic, painless build-up of pressure in the eye. This accumulation of aqueous humour causes an acute increase of pressure and pain.^4^

The primary functional and structural abnormality involved in glaucoma is glaucomatous optic neuropathy.^2^ Ocular sonography is an established method of investigating the optic nerve sheath. Previous studies have shown that there is a reduction in the optic nerve sheath diametre (ONSD) of glaucomatous eyes.^5^ The measured optic nerve sheath thickness is said to correlate well with the level of axonal loss which leads to the optic atrophy seen in glaucoma.^5^ This optic nerve damage has been shown to precede the visual field and pressure changes in glaucoma neuropathy.^5^

To reduce the burden of the disease, early diagnosis is paramount. Therefore, ocular sonography could potentially offer early and quick assessment of suspected glaucomatous eyes.

The aim of the study was to sonographically evaluate the possible changes in the ONSD and of lens thickness in patients with glaucoma in our environment.

## Materials and methods

This was a prospective, descriptive cross-sectional study carried out at the Department of Radiology of our institution over a period of one year. The Ethics and Research Committee of the hospital approved the study protocol. Written informed consent was obtained from each participant.

Adult subjects (≥ 30 years old) diagnosed with glaucoma (IOP >21mmHg and visual field showing the glaucomatous pattern as determined by an Ophthalmologist at the Ophthalmology clinic of our institution) were recruited consecutively into the study.

The control group comprised age- and sex-matched volunteers with normal IOP (<21mmHg) and normal visual field, and with no other ocular disease(s) as confirmed by the Ophthalmologist.

Exclusion criteria included: systemic hypertension (Systolic blood pressure >140mmHg and/or diastolic blood pressure > 90mmHg), diabetes mellitus (Fasting blood glucose > 5.5mmol/L), cataract, ocular trauma or history of previous ocular trauma, myopia, on-going eye infection, and previous ocular radiation therapy. Smokers were also excluded.

All the study participants were examined between 12 noon and 4pm, to eliminate the effects of circadian rhythm on intraocular pressure.

After thorough explanation of the procedure, each patient was examined in the supine position. Trans-orbital scan of both eyes was performed using a Mindray® real-time ultrasound machine model DC-7 (Shenzhen Mindray Bio-medical Electronics, Nanshan, Shenzhen, China) with a linear transducer (Frequency = 6.5 – 12 MHz). All the sonographic examinations were performed by the first author who already had 4 years' experience with ocular sonography.

Subjects' were instructed to close their eyes and copious acoustic gel was applied to the closed upper lid. The transducer was then placed on the coupling gel on the temporal region of the eye lid. Minimal pressure was applied during the examination to avoid any discomfort to the patient as well as minimizing pressure to the globe. The probe was then moved from the supero-temporal region toward the region of optic nerve entry. Proper probe angling was employed to visualize the optic nerve in its axial plane. To achieve this, patient was asked to direct his or her gaze in primary position with the probe marker centred on the cornea.

Transverse (cross-section) and longitudinal scans of both optic nerves were obtained for assessment of nerve sheath diameter. The transverse scan was done by placing the probe on the globe with the marker parallel to the limbus in two directions either nasally or superiorly. The longitudinal scan was performed by placing the probe peripheral to the limbus with the probe marker perpendicular to the limbus.

**Fig 1:**
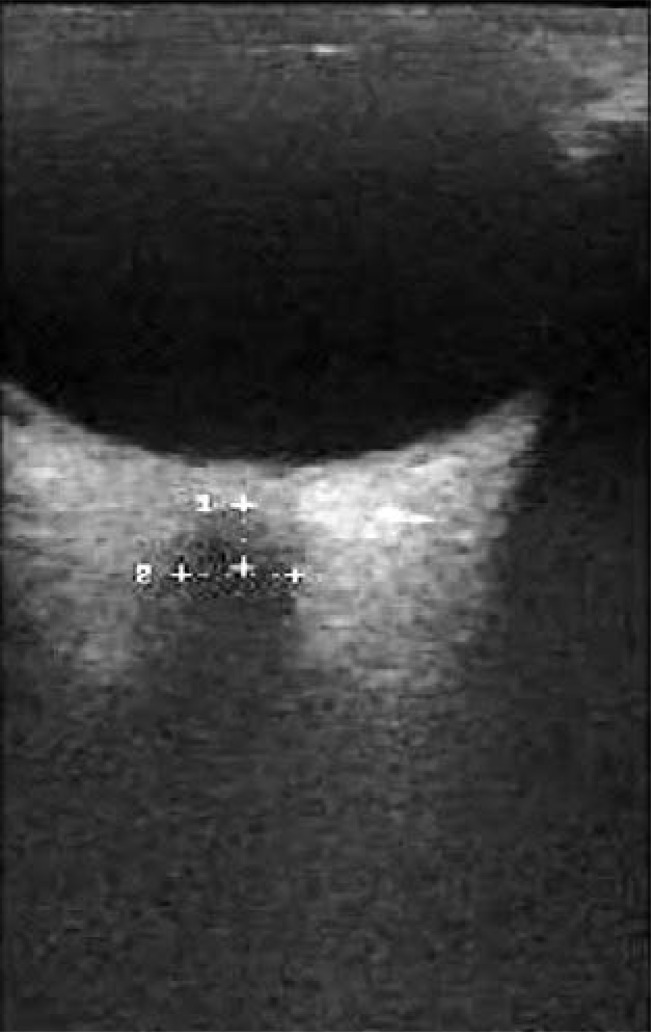


The ONSD was measured by placing the cursor on the outer contours of the dural sheath in the retrobulbar position at a distance 3mm behind the posterior wall of the eyeball (Fig. 1). The ONSD was measured as the horizontal distance between the cursors. Bilateral ONSD was measured three consecutive times and the mean value was documented.

**Fig. 2:**
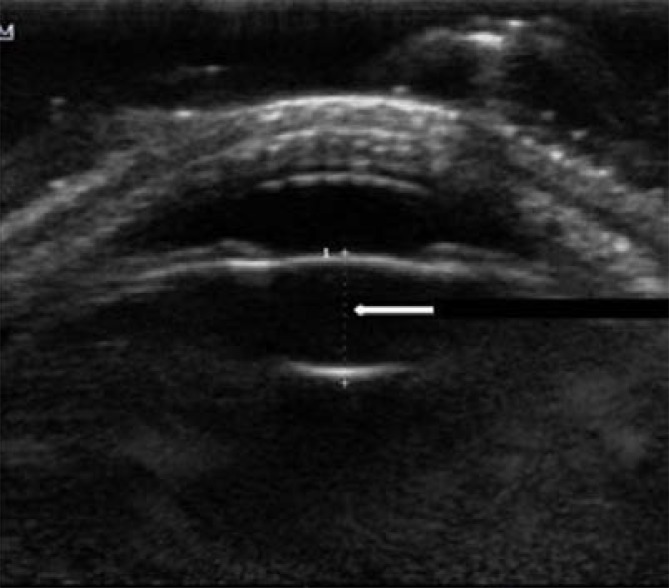
B-mode sonographic image of the eye (axial plane) showing measurement of the lens thickness (white arrow) as the anechoic space between the echogenic anterior and posterior lens capsules (delimited by cursors).

Axial lens thickness (LT) was measured by placing the cursors on the outer part of the anterior and posterior lens capsules (Fig. 2). The LT of each eye was obtained 3 consecutive times and the mean value for each eye was documented.

The data was analyzed using Statistical Package for Social Sciences (SPSS) version 18 for windows (SPSS Inc., Chicago, IL, USA). Categorical variables such as socio-demographic characteristics of respondents were presented on contingency tables for both groups, and these variables were compared between the glaucomatous and control groups using Chi-square test. Continuous variables like LT and ONSD were presented by their means and standard deviations (mean ± SD) for both glaucomatous and control groups. LT and ONSD in both groups were compared statistically using independent samples t-test. Correlational analysis was used to evaluate the relationship between age, LT and ONSD in the glaucomatous group while controlling for other variables. The relationship between IOP and ONSD in both eyes of each glaucomatous respondent were presented as categorical variable (Regular pattern or discordant) on a frequency table and analysis was done using Chi-square goodness of fit test. The level of statistical significance was set at p ≤ 0.05.

## Results

The bio-data and other characteristics of the study participants are presented in Table 1. Of the 60 glaucomatous subjects, 54 (90.0%) had POAG, 5 (8.3%) had normal tension glaucoma and 1(1.7%) had angle closure glaucoma. With the exception of weight and body mass index (BMI), there was no statistically significant difference between general characteristics of both study groups.

**Table 1:** Participants' General Characteristics

Variables	Patient category, n (%)		χ^2^	df	P value

	Glaucoma (n = 60)	Controls (n = 60)			
Age (years)					
Mean ± SD	55.5 ± 11.9	52.1 ± 11.9	1.561	118	0.121*
(Range)	33.0 –79.0	31.0 –78.0			
Gender					
Male	32 (53.3)	27 (45.0)	0.834	1	0.361
Female	28 (46.7)	33 (55.0)			
Weight (Mean ± SD) (Kg)	64.8 ± 11.2	70.3 ± 16.0	−2.320	118	0.033*
Height (Mean ± SD) (m)	1.65 ± 0.10	1.63 ± 0.08	0.717	118	0.475*
BMI (Mean ± SD) (Kg/m2)	24.10 ± 4.83	26.21 ± 5.14	−2.320	118	0.022*
FBG (Mean ± SD) (mmol/L)	3.5 ± 1.0	3.5 ± 0.9	−0.079	76	0.937*
SBP (Mean ± SD) (mmHg)	116.9 ± 13.0	121.1 ± 18.0	−1.464	118	0.146*
DBP (Mean ± SD) (mmHg)	73.5 ± 8.4	76.1 ± 10.3	−1.525	118	0.130*
Duration of glaucoma (Mean ± SD) (months)	1.2 ± 0.4	NA			

χ2 - Chi square; df - degree of freedom;

* independent samples t test applied;

SD - Standa rd deviation ; NA – Not applicable; BMI – Body mass index; FBG – Fasting blood glucose; SBP – Systolic blood pressure; DBP – Diastolic blood pressure

As expected, the mean intraocular pressure of both eyes of glaucoma subjects was significantly higher than the mean for controls (Table 2).

**Table 2:** Differences in IOP, ONSD and LT between the study groups

Variables	Participant category		t	df	P value

	Glaucoma (n = 60)	Controls (n = 60)			
Right IOP (mmHg)	17.3 ± 8.1	14.5 ± 3.1	2.560	118	0.012
Left IOP (mmHg)	17.1 ± 7.6	15.0 ± 3.3	2.034	118	0.045
Right ONSD (mm)	3.57 ± 0.19	4.23 ± 0.34	−12.881	118	< 0.001
Left ONSD (mm)	3.59 ± 0.33	4.26 ± 0.30	−11.441	118	< 0.001
Right lens thickness (mm)	4.15 ± 0.43	4.01 ± 0.56	1.615	118	0.109
Left lens thickness (mm)	4.18 ± 0.46	3.99 ± 0.45	2.285	118	0.024

IOP – Intraocular pressure, ONSD – Optic nerve sheath diametre, LT – Lens thickness, df – degree of freedom

The ONSD of the glaucomatous eyes were significantly thinner than that of the corresponding control eyes bilaterally (Table 2).

There was no statistically significant difference between the lens thickness (LT) of the right eye of glaucomatous subjects and the thickness of the right lens of controls. However, the mean LT of the glaucomatous left eyes was statistically significantly higher than that of the corresponding left eyes of controls (Table 2).

Table 3 shows that there were no statistically significant differences between the right and left eyes of glaucomatous subjects as well as the right and left eyes of controls with regard to their ONSD and LT.

**Table 3:** Paired comparison of IOP, ONSD and Lens thickness among study subjects

Variables	Mean ± SD		t*	P value

	Right	Left		
IOP (mmHg)				
*Glaucoma*	17.3 ± 8.1	17.1 ± 7.6	0.218	0.828
*Controls*	14.5 ± 3.1	15.0 ± 3.3	−2.259	0.028
ONSD (mm)				
*Glaucoma*	3.57 ± 0.19	3.59 ± 0.33	−0.513	0.610
*Controls*	4.23 ± 0.34	4.26 ± 0.30	−1.413	0.163
Lens thickness (mm)				
*Glaucoma*	4.15 ± 0.43	4.18 ± 0.46	−0.967	0.338
*Controls*	4.01 ± 0.56	3.99 ± 0.45	0.341	0.734

* Paired-samples t test

Although there was no statistically significant difference between the mean IOP of the right and left eyes of glaucomatous patients, a statistically significant difference was noted between the mean IOP of the right and left eyes of controls (Table 3).

A comparison of IOP with ONSD showed a predominant discordant pattern of relationship among glaucomatous patients in 66.7% of all cases (discordant meaning the side with higher IOP has lower or same ONSD with the contralateral side) with only about a third of glaucoma patients showing a regular pattern of relationship (regular meaning the side with higher IOP has lower ONSD) as shown in table 4.

**Table 4:** Relationship between IOP and ONSD

Variables	Patient category, *n (%)*		χ^2^	df	P value

	Glaucoma (n = 60)	Controls (n = 60)			
Discordant	40 (66.7)	29 (48.3)	4.126	1	**0.042**
Regular pattern	20 (33.3)	31 (51.7)			

Regular pattern- side with higher IOP has lower ONSD;

Discordant pattern- side with higher IOP has same or higher ONSD

Among the controls, however, there was just a marginal increase of the regular pattern over the discordant pattern (Table 4).

## Discussion

Glaucoma has consistently ranked second among the leading causes of blindness^2^,^6^,^7^ and is the leading cause of preventable, and rather unfortunately, irreversible blindness globally.^2^,^8^ The definition of this disease entity continues to evolve, allowing for continual modification of detection methods, treatment end-points and therapeutic options.^9^

Glaucoma is traditionally assessed by the triad of tonometry, visual field testing and optic nerve evaluation.^10^,^11^ However, ocular (cataracts) and non-ocular factors (physical limitations of patients) can impair the usefulness of these morphological and functional assessment methods in the evaluation of suspected glaucoma.^11^ Therefore, there arises a need to seek an alternative to this traditional triad of assessment, which will be independent of IOP and optic nerve head morphology. We surmised that since POAG is the predominant type in our environment as against primary angle closure glaucoma elsewhere, there is possibility of difference(s) in their respective ocular sonographic picture.

This study evaluated the relationship between the intraocular pressure and the size/thickness of the optic nerve sheath. The mean ONSD in glaucoma patients was statistically significantly reduced compared with the mean ONSD of healthy controls. This reduction in nerve diameter is similar to the findings of previous studies on glaucoma done elsewhere using ultrasound and Magnetic Resonance Imaging (MRI).^5^,^10^–^12^

Wang et al^5^ studied glaucomatous eyes using MRI to measure the ONSD at 3mm, 9mm and 15mm behind the eye ball. At 3mm behind the eye ball, ONSD of normal tension glaucoma (NTG) group was significantly narrower than control group. At 9mm, the ONSD of the NTG group and POAG group were both significantly narrower than the control group. At 15mm, the ONSD of the NTG group and POAG group are both significantly narrower than the control group.

Contrary to the reduction in ONSD detected in our study, Pinto et al^13^ reported no difference between the ONSD of glaucoma patients and healthy controls. Furthermore, Jaggi et al^14^ even reported an increase in the ONSD of glaucoma patients. These discrepancies in the ONSD values may reflect differences in patient selection criteria such as age, head position, sample size and most importantly, imaging modality. While ultrasound was used in this study, Jaggi et al^14^ used computed tomography and their sample size was only 18 subjects.

The mean lens thickness (LT) of the right eyes was not statistically different between the glaucoma and control groups; however, a statistically significant difference was observed on the left in this study (p = 0.024). The reason for this unilateral difference could not be ascertained in this study. From comprehensive literature search, lens thickening has been reported in closed-angle glaucomatous eyes compared to controls^2^,^15^–^19^; but this has not been hitherto documented in other forms of glaucoma.

An inverse relationship was demonstrated in this study between IOP and ONSD, with a decrease in the ONSD as the IOP increases. Comparison of ONSD and IOP in either eyes of glaucomatous subjects showed regular pattern in only a third of the patient. The vast majority of subjects with glaucoma showed the discordant pattern whereby the side with higher IOP has same or lower ONSD compared to the contralateral side. This discordant pattern was also reported by Pinto et al^13^ who did not demonstrate any correlation between IOP and ONSD in glaucomatous eyes. One possible reason for this is that, though IOP may be a risk factor in glaucoma prevalence, many cases of glaucoma may progress in spite of lowered IOP, indicating that there may be other non-IOP factors involved in the pathogenesis of POAG that can impart the apoptotic process of optic neuropathy. This was alluded to by Sommer et al.^20^

The glaucomatous optic neuropathy has been widely reported to be due to optic nerve atrophy secondary to loss of the retinal fibre layer (RNFL). It is therefore not surprising to have observed a reduction in the ONSD in this study among glaucoma patients compared with healthy eyes.

The optic nerve responds to changes in intracranial pressure because it is surrounded by cerebrospinal fluid (CSF) and meninges (Optic nerve sheath).^21^ Therefore, because the orbital CSF pressure exerts a radial stress on the optic nerve sheaths, an elevated orbital CSF pressure causes an enlargement of the optic nerve sheath which can be detected sonographically.^22^

## Conclusion

Our findings show that B-mode sonography could be an alternative tool in the assessment glaucomatous eyes as well as screening in glaucoma suspects where the traditional triad of tonometry, visual field testing and optic nerve evaluation are impossible or not available. This is very important in resource poor settings like ours.

In addition, more sonographic studies to evaluate lens thickness and other ocular biometric parameters in POAG are necessary since virtually all the previous studies evaluated primary angle-closure glaucoma.
